# Initial low‐dose oral levothyroxine in a child with Down syndrome, myxedema, and cardiogenic shock

**DOI:** 10.1002/ccr3.2169

**Published:** 2019-05-20

**Authors:** Annika Janson, Cathrin Hällström, Magnus Iversen, Mikael Finder, Amira Elimam, Ricard Nergårdh

**Affiliations:** ^1^ Department of Pediatrics Karolinska University Hospital Huddinge Huddinge Sweden; ^2^ Department of Children's and Women's Health Karolinska Institutet Solna Sweden; ^3^ Department of Intensive Care Medicine Karolinska University Hospital Huddinge Huddinge Sweden; ^4^ Department of Neonatology Karolinska University Hospital Huddinge Huddinge Sweden; ^5^ Department of Clinical Sciences, Intervention and Technology Karolinska Institutet Solna Sweden

**Keywords:** cardiogenic shock, Down syndrome, hypothyroidism, levothyroxine, myxedema, thyroxine

## Abstract

Myxedema is extremely rare in children, and guidelines are lacking. We treated a 12‐year‐old girl with myxedema and cardiogenic shock with initial low dose (0.3‐2.5 μg/kg body weight/day) of oral levothyroxine and intensive care. Oral administration may safely revert children's myxedema in a dosage resembling that for hypothyroidism.

## 1 INTRODUCTION

Hypothyroidism in children and adolescents is not uncommon,^1^, ^2^ but the propagation into a clinical emergency, *myxedema,* is extremely rare. The well‐known term *myxedema coma* refers to the altered mental state seen in myxedema. There are not precisely defined clinical criteria for myxedema coma, and it has been suggested that *myxedema crisis* better describes the life‐threatening decompensated hypothyroidism that often follows a precipitating event.^3^ This sequence has been well described in a case report of a previously healthy 5‐year‐old child admitted with altered mental status where influenza was likely to have seriously aggravated the underlying hypothyroidism.^4^

Myxedema is a condition with high mortality, 30%‐60%,^5^, ^6^ affecting adults and elderly persons more often than children. Patients present with altered mental state, low blood pressure, low pulse, low body temperature, hypoventilation, and gastrointestinal dysfunction.^5^ Pericardial effusion in children with myxedema is most often seen in children with Down syndrome.^7^

To our knowledge, guidelines on the treatment of myxedema in children are lacking and there are no controlled studies. Five published cases, including the case mentioned above,^4^ are compiled in a recent report where all children had altered mental status, and two of them had pericardial effusion.^8^ In the comprehensive guidelines from the American Thyroid Association (ATA), myxedema in adults is treated with high doses of intravenous thyroxine (200‐400 μg or higher).^9^ In contrast to the lack of guidelines for treating children with myxedema, there are well‐established guidelines on how to initiate treatment for hypothyroidism in children. Doses of oral levothyroxine (LT4) range from the 10‐15 μg/kg body weight (BW)/day used for newborn children with congenital hypothyroidism to the 4‐6 μg/kg BW/day for children aged 1‐3 years, 3‐5 μg/kg BW/day for children aged 3‐10 years, and 2‐4 μg/kg BW/day for children and adolescents aged 10‐16 years for acquired hypothyroidism.^9^

Treating patients with severe hypothyroidism has been linked to precipitating heart disease, psychiatric illness, and pseudotumour cerebri.^6^, ^10^, ^11^, ^12^, ^13^ To avoid rapid changes of the long‐standing reduced cardiac function and metabolism, severe hypothyroidism is sometimes treated with initial doses of a third to a half of usual treatment.^9^, ^14^, ^15^ It is not known to us how widespread this practice is in pediatrics. This low‐dose initiation, “start low, go slow”, is discussed in the ATA guidelines for adults and elderly people, but not for children.^9^ Obviously, centers treat children with severe hypothyroidism with full dose from the start with good outcome.^16^

There is a clinical tradition of “starting low” in treating severe hypothyroidism as well as a recommendation of high doses of intravenous thyroxine in myxedema, at least for adult patients. Neither strategy is supported by controlled studies and the balance of risk and benefit may not be the same for children as for adults and elderly persons. Also, some existing reports on myxedema are old, thereby lacking access to the benefits of modern intensive care.

To our knowledge, there are only two very recent reports on children with myxedema treated with oral preparations, and neither was published to guide us at the time our patient was treated. Zhu et al successfully treated a 6‐year‐old child with an initial dose of 4 μg/kg/day,^17^ and Wankanit et al treated a 2‐year‐old patient with 6.8‐7.3 μg/kg/day.^8^ The remaining four patients in the review by Wankanit were successfully treated with intravenous thyroxine in doses 3‐10 μg/kg/day.^8^

## 2 CASE HISTORY

A 12‐year‐old girl, the youngest of eight siblings, who had arrived to Sweden four days earlier from Afghanistan, was admitted to the emergency room (ER). She was conscious but confused and in cardio‐respiratory shock with low saturation (60%), prolonged capillary refill (4 seconds), high temperature (39°C), hypotonia, and bradycardia (50 beats/minutes). She had a marked generalized and dense swelling (abdomen, arms, face, tongue, and skin) with skin‐colored nodules on arms and legs.

Down syndrome had been suspected by the parents but the girl had not been investigated in Afghanistan. The family described a normal birthweight with neonatal hypotonia and reported that the girl had been healthy but with delayed motor development, walking at 3 years of age, and delayed intellectual development and no speech. She had not attended school. She had gradually lost vigor, gained weight, and developed a coarse skin over the last months or year prior to this emergency. However, she had been traveling by air, walking, and interacting with people just a few days before admission. Despite modest development of secondary sexual characteristics, the family reported that she had menstruations. Three days prior to the hospitalization, the girl fell sick with fever and cough and deteriorated rapidly. The girl's altered mental status and generalized weakness prompted the family to search immediate care.

In the ER, the patient was intubated, sedated, and ventilated by hand and received repeated intravenous bolus doses of fluid. One near‐cardiac arrest was overcome with ventilation with high pressures. Pericardial effusion was observed on echocardiography, and 130 mL fluid was evacuated with a drainage left in the pericardium to evacuate fluid over the coming days.

## 3 DIFFERENTIAL DIAGNOSIS

The patient was suspected of having a septic shock and pericarditis and was given full intensive care with antibiotics and inotropic and sedative drugs. One single dose of steroid, 10 mg prednisolone, was given in the ER Mucopolysaccharidosis was briefly considered to be an underlying diagnosis, and a resemblance of features of Down syndrome was also noted.

## 4 INVESTIGATIONS AND TREATMENT

C‐reactive protein was 58 mg/L (ref <3). No infectious agent apart from metapneumovirus was identified. The patient demanded high pressures in the respirator with up to 70% oxygen, and bowel movements were very limited with severe constipation and subileus. On day 3, the patient was transferred to our hospital and ICU, still having generalized swelling. She had bradycardia (50‐60/min), hypotension (mean arterial pressure 60 mm Hg), and normal temperature. The thyroid was not clearly enlarged but difficult to examine due to the generalized nonpitting edema and the presence of a tracheal tube and a cervical artery needle. The height was 130 cm (−4 SD).

**Figure 1:**
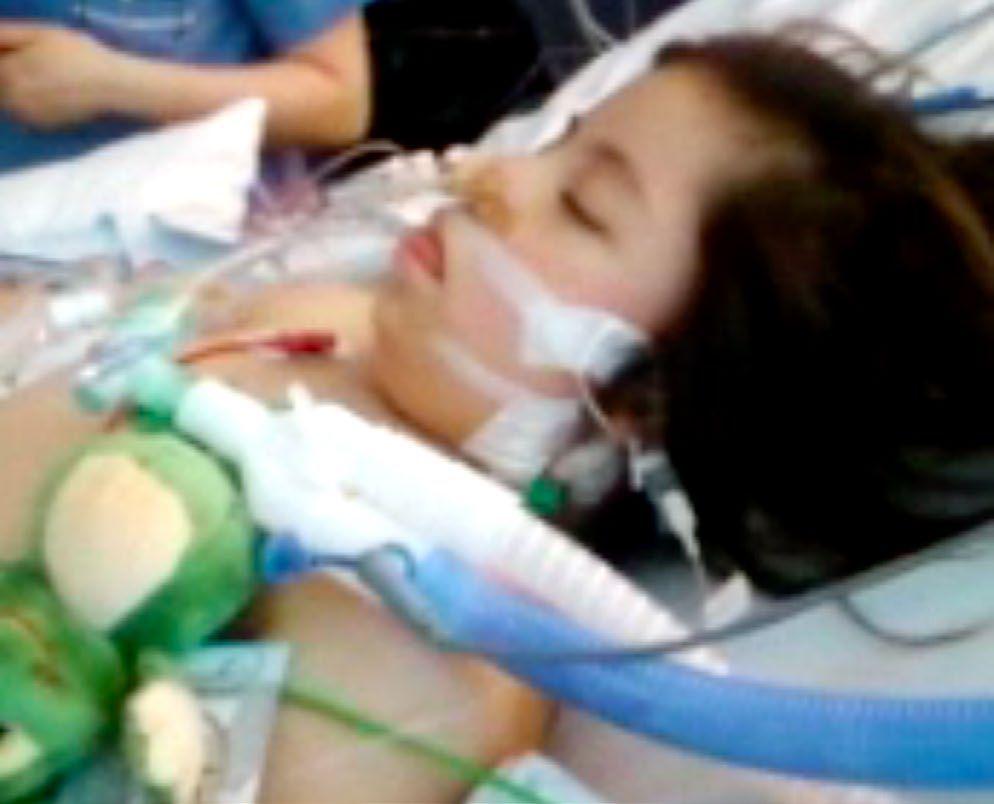
Patient critically ill at the time of start of treatment of myxedema on day 4

Myxedema was suspected on day 3 and confirmed with laboratory tests. Figure 1 shows patient at start of treatment. Thyrotropin (TSH) was 346 mU/L before start of treatment (reference 0.4‐4.7), and free thyroxine (fT4) was 1.4 pmol/L (reference 12‐23). Antibodies to thyroid peroxidase were positive in a high titer, 106 kE/L (reference < 34), and Hashimoto's disease (Hashimoto's thyroiditis) was diagnosed. Antibodies to thyroglobulin were slightly elevated, 59 μg/L (reference < 55). Serum levels of sodium and potassium were normal and initial cortisol level was not measured, but s‐cortisol was measured on day 6 and was normal, 531 nmol/L (reference 200‐800). Hemoglobin was 103 g/L (reference 110‐160), platelet count 105 × 10^9^/L (reference 150‐400), liver enzyme aspartate aminotransferase was elevated, and serum creatinine was slightly elevated.

Treatment with oral LT4 was started with very low initial doses and using a nasogastric tube for administrating the crushed tablets (Table 1). The rationale for using oral preparations was in part practical, the unavailability of intravenous thyroxine. The rationale for choice of doses were based on the patient being critically ill, but stable, in intensive care and our previous clinical experience of treating severe hypothyroidism with initial low doses. Daily laboratory tests guided further choice of daily dosage of LT4 where we aimed for a slow normalization and adjusted daily doses to see a slow increase in fT4 (Table 1).

**Table 1:** Thyrotropin (TSH), free thyroxine (fT4), and free triiodothyronine (fT3) (normal references in brackets) and the selected dose of oral levothyroxine (LT4) for the first ten days of treatment and at discharge on day 28

Day^a^	TSH (0.4‐4.7 mU/L)	fT4 (12‐23 pmol/L) *(0.9‐1.8 ng/dL)*	fT3 (3.0‐6.5 pmol/L) *(0.2‐0.4 ng/dL)*	Dose of LT4 (μg)	Dose of LT4 (μg/kg BW^b^)
4	346	1.4 *0.11*	1.4 *0.09*	12.5	0.29
5	487	1.4 *0.11*	<1.0 *<0.06*	25	0.57
6	>500	2.2 *0.17*	1.0 *0.06*	25	0.57
7	412	2.0 *0.16*	1.1 *0.07*	100	2.3
8	416	3.2 *0.25*	1.1 *0.07*	50	1.15
9	426	3.3 *0.26*	1.2 *0.08*	50	1.15
10	‐	‐	‐	125	2.9
11	421	‐	‐	100	2.3
12	334	7.0 *0.55*	1.4 *0.09*	50	1.15
13	332	‐	‐	75	1.75
14	286	8.2 *0.64*	1.9 *0.12*	75	1.75
28	92	24 *1.88*	4.5 *0.29*	50	1.29

a Day 0 is day of admission.

b Body weight (BW) was measured on day 4 and 28 only.

## 5 OUTCOME AND FOLLOW‐UP

The clinical suspicion of Down syndrome was confirmed with genetic analysis that showed trisomy of chromosome 21. Apart from autoimmune Hashimoto's disease, the patient was also diagnosed with celiac disease with transglutaminase IgA antibodies 75 E/mL (reference < 7). Also, the diagnosis of permanent type‐1 diabetes evolved during the hospital period, with glutamic acid decarboxylase antibodies positive in a high titer, >250 E/mL (reference <5).

**Figure 2:**
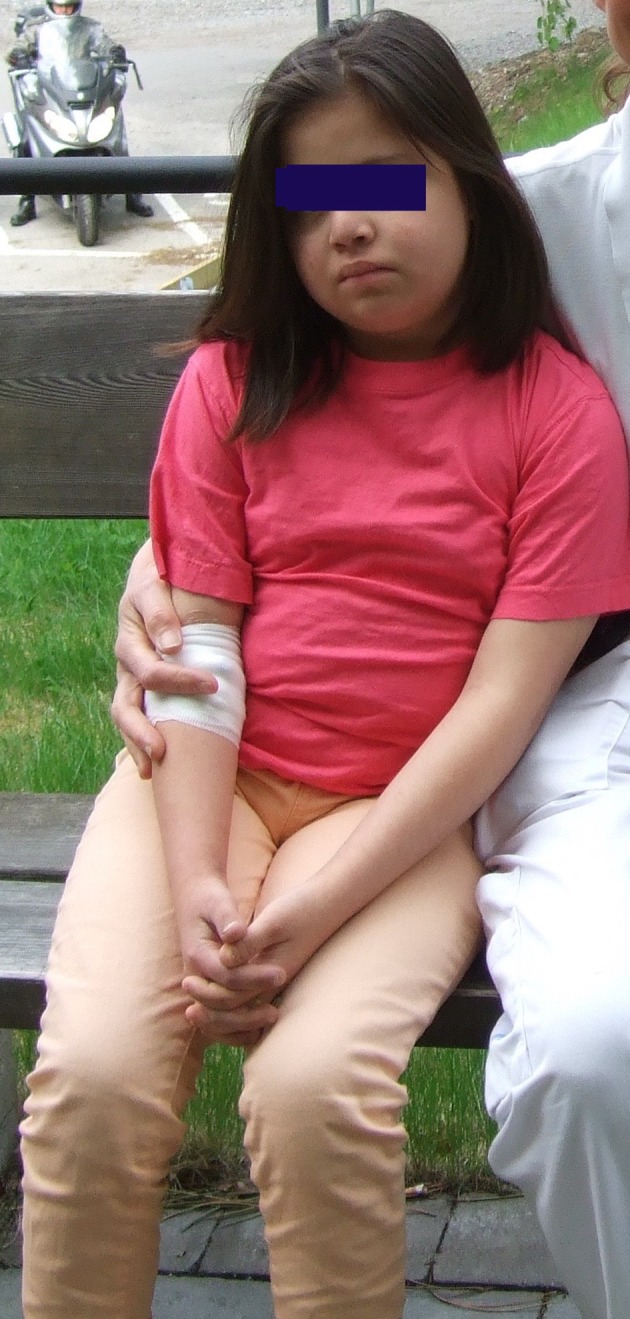
Patient at discharge on day 29 with diagnoses of Down syndrome, Hashimoto's disease, diabetes type 1, and celiac disease

The patient was treated in a respirator for 15 days with sedative drugs gradually lowered, so that she was awake on day 9 onward. She was then alert, accepted the respirator, recognized her relatives, and communicated with signs. On day 12, seizures (rolling of eyes) were suspected and midazolam was given. Following the suspected seizures, a 2‐day continuous EEG registration showed a slight to moderately pathological generalized slow activity but no seizure activity. Oral steroids were given to assist at the time of extubation and then gradually lowered over the next three weeks. The patient stayed in the hospital for a total of 29 days. Repeated echocardiography revealed no structural heart anomaly. Ultrasound of the thyroid gland on day 7 showed normal sized thyroid gland, 18 × 7 × 7 mm. BW prior to diagnosis was 43.5 kg, and 38.4 kg on discharge. Figure 2 shows patient at discharge.

The long‐term outcome has been successful. Five years later the girl has had a growth spurt of 12 cm with a final height 142 cm which is −1 SD on the Swedish growth chart for children with Down syndrome.^18^ She has regular menstruations, lives with her family and goes to a special school. She can express herself with a limited number of words, sounds, and signs and has the habitus of a Down syndrome patient. She is treated with LT4 (100 μg/day), insulin (approximately 0.7 U/kg BW/day), and gluten‐free diet.

## 6 DISCUSSION

Myxedema is a rare condition and an emergency with high mortality. This likely contributes to the lack of randomized studies of patients of any age, or reports on treatment of groups of children to aid clinicians in these desperate situations. One controversy is whether to use high starting doses of thyroid hormones, or considerably lower doses.^9^ Secondly, intravenous administration is recommended as uptake can be compromised by intestinal edema, but other authors argue that oral preparations work, also in children.^8^, ^17^, ^19^ Thirdly, liothyronine (LT3) is often recommended along with LT4 as the peripheral conversion of LT4 into the more active form LT3 is believed to be compromised as well.^9^, ^20^

The issue of initial dose was discussed in the 1980s by Hylander and coworkers who demonstrated in a study on 14 hypothyroid adults that the ability to increase pulse rate in an orthostatic test recovered earlier than the ability to perform cardiac work in an exercise test, suggesting that some effects are slower to normalize following substitution with LT4 than others.^21^ In another study of eight adult patients with myxedema, the authors observed that fatal outcome was more common among the patients treated with higher doses of LT4 and the authors made the same observation in an additional analysis of 82 patients from the literature.^11^ In a comment to a case report^4^ that further illustrates the importance of considering the long‐term hypothyroid state in various organs, the author points out that also the gut may be slower to recover from the long‐standing hypothyroid state which may affect the time needed for intravenous nutrition.^22^

However, the clinical tradition of “starting low” and avoiding rapid changes to the hypothyroid state, has been challenged in one prospective randomized double‐blind trial of 50 adults with hypothyroidism without cardiac symptoms where a full starting dose of 1.6 μg/kg BW to patients with mean TSH of 61 mU/L (range 14‐797) was considered safe and lead to faster normalization of thyroid status in comparison to a low starting dose of 25 μg.^23^

It has been questioned whether complications arise because of the disease or the treatment. In one case, a 7‐year‐old girl with congenital hypothyroidism and poor compliance developed pericardial and bilateral pleural effusions one week after the reintroduction of LT4 with a dose of 100 μg daily.^7^

Another reason for low starting doses of LT4 has been the association of pseudotumor cerebri with the onset of treatment,^13^ but pseudotumor cerebri has been noticed also in children where the low dose was used.^24^ In one study of three adult cases, intravenous LT3 reverted the situation for one of the patients that had failed to respond to oral LT3 treatment.^20^ The use of LT3 has, however, been considered to have a greater risk of fatal outcome.^6^

In our case, intravenous LT4 or any LT3 was simply not available within a reasonable time. We used oral LT4 and started with a low dose. We followed daily TSH, fT4, and fT3 and adjusted the LT4 doses daily. The initial dosage was likely too low and was increased (Table 1). Still, the dosage is in the vicinity of the recommended dose of 2 μg/kg for children in this age when treated for common hypothyroidism. Likewise, the doses used by Zhu et al who used 4 μg/kg/day for a 6‐year‐old child 17 and Wankanit et al 8 who used around 7 μg/kg/day for a 2‐year‐old child are similar to the dosage recommended for the ages of the children they treated, and slightly higher than the one we initially chose.

In Sweden, national guidelines recommend for children with Down syndrome to be screened at regular intervals as the risk for hypothyroidism and other autoimmune diseases is increased but this patient did not live in Sweden prior to her emergency.^25^ In our patient, clinical improvement was slow but apparent from start of treatment and the long‐term outcome was successful. In conclusion, in the presence of modern intensive care, it seems safe to avoid high doses of intravenous thyroxine and instead treat children with myxedema with oral thyroxine in doses resembling that for a child of similar age with hypothyroidism.

## CONFLICTS OF INTEREST

The authors have no financial relationships relevant to this article. No competing financial interests exist. No specific support has been given for this work. The authors have no conflicts of interest relevant to this article to disclose.

## AUTHOR CONTRIBUTION

AJ: wrote the paper with input from the other authors. All the authors participated in the clinical care of the patient at the time.
